# Comment on ‘‘Elucidating the binding efficacy of *β*-galactosidase on graphene by docking approach and its potential application in galacto-oligosaccharide production”

**DOI:** 10.1007/s00449-017-1736-8

**Published:** 2017-01-31

**Authors:** Marek Wiśniewski, Katarzyna Roszek

**Affiliations:** 1 Faculty of Chemistry, Physicochemistry of Carbon Materials Research Group, Nicolaus Copernicus University in Toruń, Gagarin Street 7, 87-100 Toruń, Poland; 2 INVEST-TECH R&D Center, Plaska St. 32-34, 87-100 Toruń, Poland; 3 Department of Biochemistry, Faculty of Biology and Environment Protection, Nicolaus Copernicus University in Toruń, Gagarin Street 7, 87-100 Toruń, Poland

**Keywords:** Immobilization, *β*-Galactosidase, Graphene, Graphene oxide

## Abstract

In a recent study, Satar et al. (Bioprocess Biosyst Eng 39:807–814, 2016) have reported “the synthesis and characterization of graphene for the immobilization of *β*-galactosidase for improved galacto-oligosaccharide (GOS) production”. There are several issues in this study that must be commented.

The recently published work by Satar et al. [1] concerns the study of enzymatic activity and stability of immobilized *β*-galactosidase.

At first, it is necessary to address the problem of nomenclature, i.e., graphene and graphene/graphite oxide. Authors use these terms interchangeably which are highly misleading. Authors use “graphene”; however, they synthesized graphene oxide (in fact graphite oxide). Already in “Introduction”, graphene properties are described instead of quite different materials which were synthesized, i.e., graphene oxide (GO). The discrepancy lies in the fact that GO does not possess only sp^2^ bonded carbon atoms. Moreover, it exhibited neither high thermal conductivity nor high-specific surface area as graphene. In addition, in Fig. 2, the scheme is related to graphene oxide not graphene. In Fig. 1, one can find “Transmission electron microscopy revealing the size of graphene”. There are two problems: (1) it is highly improbable to achieve such perfect surface of graphene oxide (it is not a TEM picture) and (2) this picture does not reveal the size of graphene. In our opinion, the presented graphene sheet is generated in silico.

Misrepresentation of the experimental parts has become common in recent years, so it is not worth mentioning that the synthesis cannot be performed as it is described. Nevertheless, in the cited reference, the method of GO production proposed by Zhao et al. [2] is completely different. Mixture obtained during the oxidation, in accordance with the recipe, is too viscous for mixing, what results in an incomplete reaction. In addition, the amount of oxidant appears far too small per 1 g of graphite, thus causing that the final product is far from yellow (after 24 h reaction).

However, more important shortcomings are presented further in this section:

- How is it possible to force ethanol to dissociation through simple bath in GO solution? Even if so, authors did not prove this. Moreover, what is the sense of incorporation of –OH into the material which possesses lots of such functionalities?
- The concentrations of GO at the level of few mg/mL cause that the solution possess very high viscosity. Thus, reasonably seems to be using 1 mg of GO. Nevertheless, it would be helpful to know how much enzyme was used, as well as the volume of the solution.
- “*β*-Galactosidase was stirred slowly in modified GO (1.0 g) and kept for overnight”—the authors provide no information about the concentration/amount of protein added, no immobilization yield (was the whole protein adsorbed? How much enzyme was unbound and discarded?)
- The yield calculated by authors (from equation) is nowhere mentioned in the manuscript. Why?

Another chemical problem appears in “enzyme immobilization”. Authors unnecessarily describe it (and shown in Fig. 2) as covalent bonding.

The principle of direct alkylation of amines with alcohols, leading to the formation of the secondary amines from primary ones, is known in the literature [e.g., 3]. However, the N-alkylation reaction usually needs metal catalyst. In addition, the N-arylation is not possible under conditions mentioned in the article. Furthermore, there is no proof, in this work, of this hypothesis. It is well known that on the surface of graphene oxide proteins are very strongly adsorbed physically. The interactions are strong enough to keep immobilized enzyme particles on the GO surface during washing it with buffer.

Extremely huge problem is with Fig. 3. In fact, this figure shows completely nothing and it was the inspiration for this comment. Let us first start from “Experiment”, and actually the lack of it. It is not so important to write the name of FTIR apparatus, as the obtained spectrum should be independent on it. More important is to describe the used technique, ATR, DRIFT, transmittance, etc. From “The nondestructive analysis”, readers know essentially nothing.

The spectra in Fig. 3 are of extremely weak quality. Skipping the fact that there is no scale, and usually, it is impossible to obtain in transmittance mode the up-going bands (except for the differential spectrum; however, these were not the intention of authors). The bands “present at 3105 cm^− 1^”: (1) cannot be attributed to –OH and (2) there is not such a band in Fig. 3. Similarly, “the broadening of peaks from 1200 to 1700 cm^− 1^”: (1) there are no such a peaks and (2) even if so, such effect cannot be attributed to protein attachment to the GO.

Problem with Figs. 4 and 5 and their description is that authors did not realize the real size of the graphene sheet. Omitting the fact that it is not a graphene oxide, the size of modelled sheet is extremely too small.

Summarizing, the analysis of catalytic activity was done on graphene oxide, not activated GO nor graphene. The material was not characterized and “The size of synthesized graphene was observed to be 25 nm by TEM analysis while interaction of enzyme with the nanosupport was observed by FTIR spectroscopy.” This sentence from Abstract is untruth. As we fully agree with authors’ statements written in the “Conclusion”, it is necessary to mention that (1) claim “Immobilized *β*-galactosidase (IβG) showed improved stability against various physical and chemical denaturants” was not proved here; authors tested only the influence of the temperature and (2) these are not the conclusions, rather a summary.
